# Impacts of Pre-bloom Leaf Removal on Wine Grape Production and Quality Parameters: A Systematic Review and Meta-Analysis

**DOI:** 10.3389/fpls.2020.621585

**Published:** 2021-02-04

**Authors:** Joshua VanderWeide, Chris Gottschalk, Steven R. Schultze, Esmaeil Nasrollahiazar, Stefano Poni, Paolo Sabbatini

**Affiliations:** ^1^Department of Horticulture, Michigan State University, East Lansing, MI, United States; ^2^Faculty of Land and Food Systems, Wine Research Center, The University of British Columbia, Vancouver, BC, Canada; ^3^Department of Earth Sciences, University of South Alabama, Mobile, AL, United States; ^4^Michigan State University Extension, East Lansing, MI, United States; ^5^Department of Sustainable Crop Production, Università Cattolica del Sacro Cuore, Piacenza, Italy

**Keywords:** bunch rot, canopy management, defoliation, fruit quality, grapevine, rootstock

## Abstract

Wine grape (*Vitis vinifera* L.) is the most widely cultivated fruit crop in the world. However, the climactic characteristics in some growing regions are suboptimal for grape production, including short season length and excess precipitation. Grape growers can utilize an array of methods to mitigate these issues, including “early leaf removal,” a management practice involving the removal of leaves from selected basal nodes along shoots around bloom. This meta-analysis reviews the extensive literature on this practice, with specific regards to application at “pre-bloom” (PB). One hundred seventy-five publications on the topic of “early leaf removal” were identified using key terms and subsequently narrowed via eight data curation steps. The comparison between treated (PB) and control plants in these studies revealed two important results. First, PB lowered bunch rot disease (−61%), partially through reducing the compactness of clusters. Second, PB promoted a significant increase in fruit total soluble solids (°Brix, +5.2%), which was related to the increase in the leaf-to-fruit ratio. Furthermore, cultivar and rootstock were found to have a large influence on the success of PB, while the contribution of climate was smaller. In conclusion, PB significantly lowers yield and bunch rot disease and increases °Brix, both of which improve grape and wine quality.

## Highlight

- A meta-analysis of 59 publications revealed that the wine grape management practice “pre-bloom leaf removal” consistently decreased bunch rot disease, yield, and cluster compactness while improving fruit sugar concentrations.

## Introduction

Grapevines are among the most intricately managed food crops due to their sensitivity to external and internal factors, such as the environment and source–sink relations (Kliewer and Dokoozlian, 2005). The interaction between internal and external factors has given rise to the notion of “terroir,” unique to viticulture and enology (Van Leeuwen, 2010). Several viticultural practices are utilized to align vine growth, vine development, and fruit ripening (internal factors) with environment conditions (external factors). One such practice is “leaf removal,” otherwise referred to as “defoliation” or “leaf thinning.” Leaf removal is a technique that involves the removal of a select number of leaves that cover the fruiting region along shoots (Poni et al., 2006). This allows for a more open fruit-zone microclimate, which can lead to numerous production and fruit quality benefits.

Using the Eichhorn-Lorenz grape phenology scale as a reference (Coombe, 1995), the two most researched times of leaf removal application are (1) “early,” which includes application from “pre-bloom” (E-L 17, flower caps on) through “bloom” (E-L 23, flower caps off) and “fruit set” (E-L 27, berries >2 mm), as well as (2) “late,” which centers around “veraison” (E-L 35, berry ripening initiation).

The primary objective of early leaf removal practices is the mitigation of yield loss from cluster rot diseases, such as gray mold (*Botrytis cinerea*) and sour rot, particularly in compacted cluster varieties (Poni et al., 2017). In warm/hot, dry growing regions, gray mold is more prominent. Gray mold is a necrotrophic fungus ubiquitous to crops and particularly fruit production (Ky et al., 2012). It initially infects fruit from the surface, followed by degradation of subtending tissues, leading to a loss of yield while compromising quality-related metabolites, such as organic acids, phenolics, and volatiles. In cool/warm regions that receive high volumes of precipitation during the fruit ripening period, sour rot is the more problematic form of bunch rot disease. The bacteria and yeast comprising the sour rot complex convert the fruit sugars (glucose, fructose) into acetic acid and other metabolites, such as acetaldehyde, galacturonic acid, gluconic acid, ethanol, ethyl acetate, and glycerol (Zoecklein et al., 1995). Increases in the concentration of acetic acid engenders a noticeable “vinegar” flavor to wines made from these fruits, thus lowering quality and value.

The second major objective of early leaf removal is to enhance fruit and wine quality (Tardaguila et al., 2010; VanderWeide et al., 2018). Crop load regulation is required in specific regions to meet yield standards in some prominent production regions, such DOCG in Italy or AOC in France. Additionally, in warm/hot, dry growing regions, the yield of highly fruitful cultivars must be reduced to maintain vine balance, and early leaf removal provides an effective tool to achieve targeted crop levels. This, in turn, leads to an improvement in both basic fruit quality components as well as total anthocyanins (Tardaguila et al., 2012; Poni and Gatti, 2017; Silvestroni et al., 2018). In addition to crop level, the capacity of a grapevine to produce “high-quality” fruit is related to seasonal accumulation of growing degree days (GDDs). Cool/warm regions are defined by low mean day temperatures, while the low GDDs experienced by vineyards in cool regions can also hinder the accumulation of hexoses in fruit (Liang et al., 2014).

Leaf removal at pre-bloom consistently induces a reduction in fruit set in both red and white cultivars (Poni et al., 2009; Sabbatini and Howell, 2010; Tardaguila et al., 2010; Molitor et al., 2011; Acimovic et al., 2016). Carbon deprivation from leaf removal at this stage impacts meiosis in inflorescence, reducing the flow of hexoses and decreasing flower fertility (Lebon et al., 2004). The severity of leaf removal at either pre- or after-bloom greatly affects fruit set, as well as developmental processes throughout fruit ripening. Using Pinot noir (*Vitis vinifera* L.), Acimovic et al. (2016) evaluated the response of removing 4, 6, 8, or 10 leaves. They reported that the removal of six and eight leaves only induced the desired effect on reducing fruit set and improving fruit quality. Removal of 4 leaves had little to no effect, while 10 leaves induced a severe carbon stress on vines, decreasing yield below an economical viable threshold (Acimovic et al., 2016). This decrease in fruit set lowers the compactness of clusters, which has significant impact on gray mold (Gubler et al., 1991; Palliotti et al., 2011; Sivilotti et al., 2016) and sour rot (Zoecklein et al., 2000; Mosetti et al., 2016; Sivilotti et al., 2016).

An increase in total soluble solids (TSS) was observed in fruit subjected to pre-bloom leaf removal when compared to the undefoliated control (Poni et al., 2006; Zenoni et al., 2017), while some results were mixed between treatments and years (Acimovic et al., 2016). Mixed results were seen for alterations in pH and titratable acidity (Intrieri et al., 2008; Acimovic et al., 2016; Zenoni et al., 2017). Pre-bloom leaf removal's effect on total phenolics is inconsistent, with some studies observing a consistent increase compared to the control (Poni et al., 2006; Intrieri et al., 2008) and others reporting no differences (Talaverano et al., 2016). The majority of publications reported an increase in anthocyanins with pre-bloom leaf removal compared to the control (Poni et al., 2006; Lee and Skinkis, 2013; Pastore et al., 2013; Zenoni et al., 2017), while some results were mixed between years, treatments, or varieties (Tardaguila et al., 2010), and some reporting no differences in all years and treatments of experimentation (Lee and Skinkis, 2013; Acimovic et al., 2016; Sivilotti et al., 2016).

Previous reviews in viticulture have focused on grapevine management practices (Smart, 1985), with some devoting space to this practice (Poni et al., 2017). Still others have reviewed the practice of early leaf removal within a specific region (Verdenal et al., 2019) or with a particular focus on aroma biosynthesis (Wang et al., 2018; Alem et al., 2019). However, no review or meta-analysis has been published in the literature that approaches the impact of early leaf removal on major production and quality traits. The objectives of this meta-analysis were 2-fold. The first objective was to understand whether pre-bloom leaf removal has a consistent impact on production and fruit quality parameters regardless of differences in climate, cultivar, rootstock, vine age, or berry color. The second objective was to assess whether factors, such as climate, cultivar, rootstock, vine age, or berry color influence the success of pre-bloom leaf removal on production and fruit quality parameters. This meta-analysis seeks to confirm the collective hypotheses generated from publications in this field in order to direct future research.

## Materials and Methods

### Data Collection

A literature review was performed to identify works published from January 1985 to May 2020 in peer-reviewed scientific journals and conference proceedings that focused on the topic of early leaf removal in grape. MS Thesis and Ph.D. Dissertations were not included. We used search terms of “defoliation grape” and “leaf removal grape” in Google Scholar and Web of Science to identify works for inclusion. A total of 175 publications were identified that involved the removal of leaves in grape.

### Data Curation

**Figure 1:**
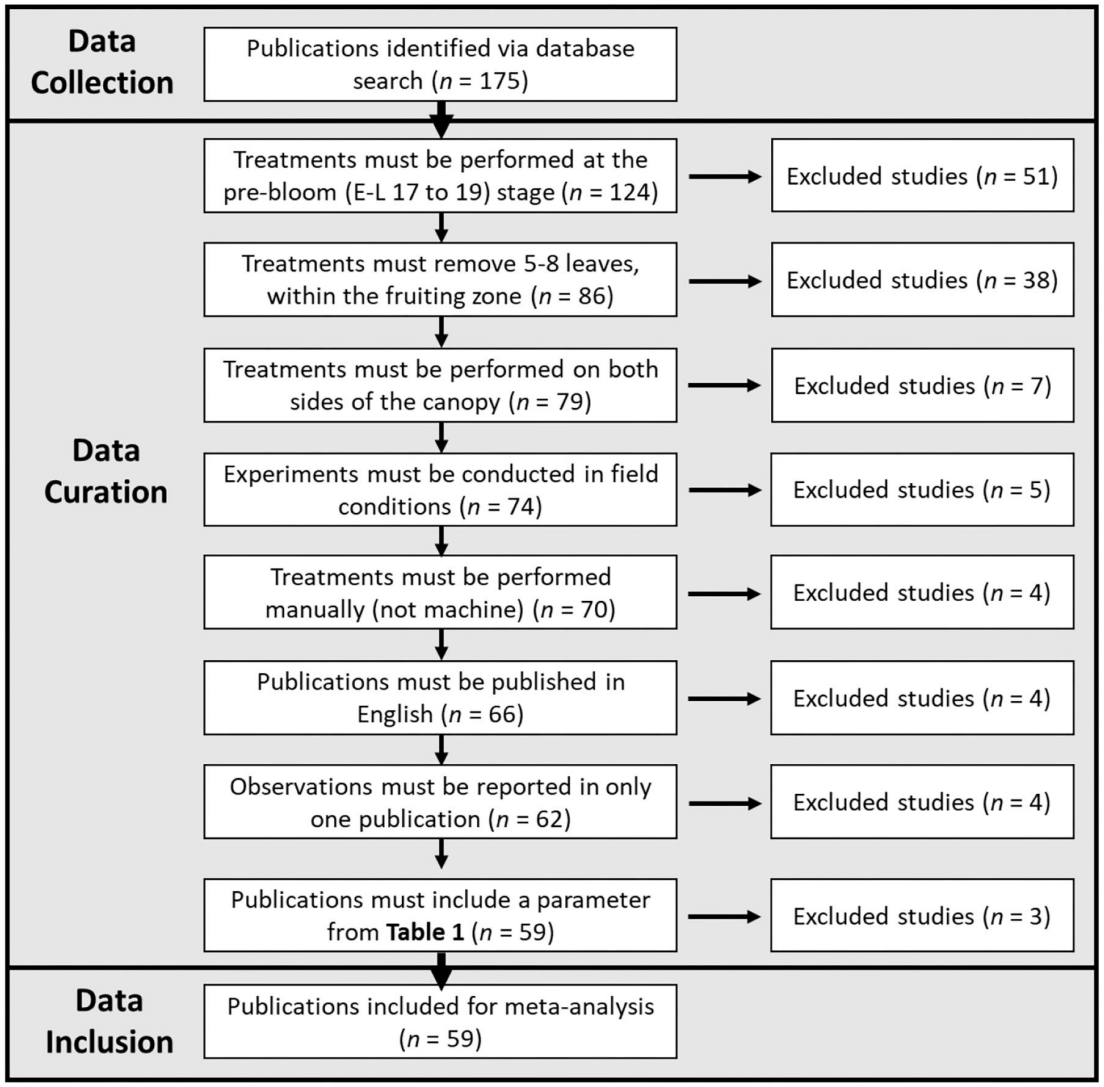
Flowchart demonstrating the data collection, data curation, and data inclusion process utilized in this meta-analysis.

Publications were maintained for further statistical analysis according to Figure 1.

The exclusion of publications to fit these seven criteria resulted in 59 studies (Supplementary Figure 1). In some cases, data from the same experiment (observation/s) were presented in multiple publications, and when this occurred, the duplicate/s of these data were eliminated from analysis. In cases where all desirable data from a study was present in a previous publication, the more recent study was excluded. “Training system” and “Species” were originally considered as categorical variables; however, only two publications in our curated set included vines not trained to a vertical shoot positioning trellis system and two with vines that were not *vinifera* species, so they were maintained without further categorization. For each publication, in case that desired data were only present in figures, ImageJ software (Version 1.51e) was utilized to extract data points when the treatments from the respective publication were distinguishable. In the case of “yield,” “cluster compactness index,” “bunch rot incidence,” “bunch rot severity,” “total anthocyanins,” and “total phenolics,” unit representation of some parameters was heterogeneous between studies. When possible, data were converted to a common unit. For “yield,” “shoot number per vine” data were used to convert “yield/shoot” to “yield/vine,” and “vine density” data were used to convert “yield/meter (row length)” to “yield/vine.” In the case of “total anthocyanins” and “total phenolics,” data were converted to “mg/100 g (fresh weight).” In the case that multiple acceptable units were presented in a publication, all were included. Such was the case only for “cluster compactness index” and “bunch rot incidence/severity.” In two instances, severe outliers that could be attributed to a miscalculation in the publication were deleted prior to analysis. This was the case for “total phenolics (mg/100 g) FW berry” (VanderWeide et al., 2018) and “total phenolics (AU)” (Frioni et al., 2018).

### Climate Data

Thirty years climatological normals data were obtained from several meteorological agencies with long-term, monthly climate normals for temperature and precipitation (NCDC 2020, MeteoSwiss 2020, DataMeteo 2020, Agencia Estatal de Meteorologia 2020, Hydrological and Meteorological Service of Montenegro 2020) for each location included in this study. In most cases, weather data were available for the study location. However, there were a few locations that did not have data, as the location was not located in a specific “town.” As such, the nearest station with similar conditions (elevation, windward/leeward dynamics) was used. The alternative stations were never more than 15 km away from the research location.

The climate data obtained allowed us to separate observations into four types: Climate 1 (hot), Climate 2 (warm/dry), Climate 3 (warm/wet), and Climate 4 (cool). The delineations between each cluster were based on average growing season temperature (GST) and average total precipitation. Climate 1 points included all study locations with average GSTs above 20°C. Climate 4 points included all study locations with average GSTs below 16°C. Climates 2 and 3 have temperatures between 16 and 20°C and are delineated by having more or <500 mm precipitation (the median for all location precipitations was 462 mm). It should be noted that these delineations serve as cutoffs for the data points we have acquired for this study. Temperature is based roughly on the breakdown of climatic classes by Jones (2007). The 500-mm precipitation cutoff for Climates 2 and 3 exists to differentiate between the largest pool of climates (38). This cutoff was deemed necessary because, if it did not exist, this study would consider Oslavia, Italy (17.9°C, 851 mm) the same climate classification as Erzcinan, Turkey (17°C, 187 mm).

### Statistical Analysis

**Figure 2:**
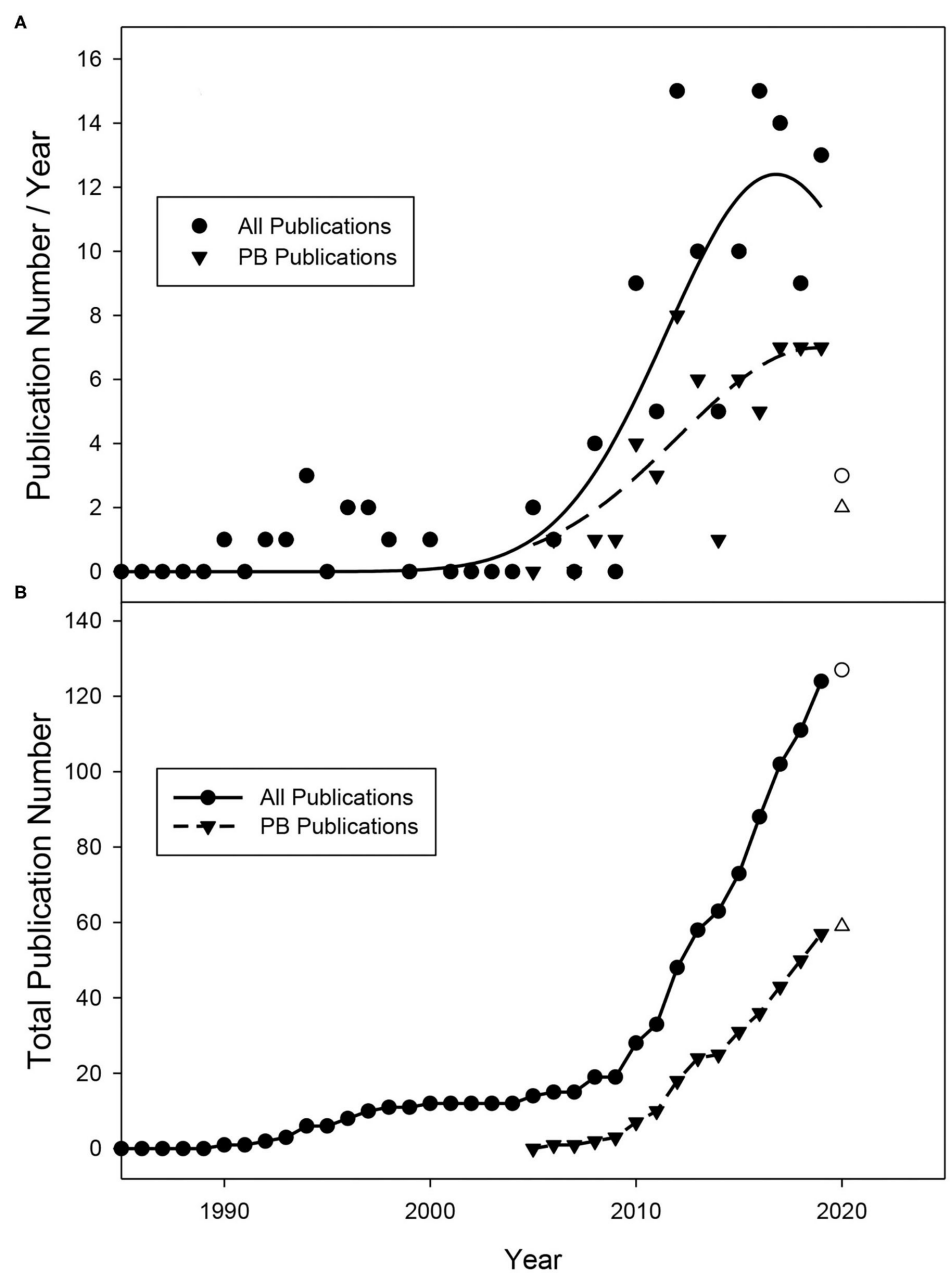
**(A)** Publication number per year and **(B)** total publication number obtained from database searches between January 1985 and May 2019. No publications were identified prior to 1988. Data from 2020 (hollow circle) does not encompass the entire year (January–May) and is not included in regression analysis.

Among the 59 publications used for analysis, few reported the standard error for all the parameters included in this study. Given this, the variable errors within each experiment were not accounted for. For all dependent variables, power was calculated using the G^*^Power Software (version 3.1.9.7). For dependent variables (Supplementary Table 2, **Figures 4**–**6**), an independent samples *t*-test (*p* = 0.05) was used to compare pre-bloom leaf removal treatments against the untreated control using IBM SPSS software (IBM, Armonk, NY, USA). When parameters were expressed as a percentage, the multiple acceptable units for each parameter were combined. In the case that multiple forms of a parameter existed in a publication (“cluster compactness index,” “bunch rot incidence/severity”), both were included, and the data from the remaining parameters doubled. Factor analysis of mixed data (FAMD) was conducted using R version 3.6.2 (R Core Team, 2016). For FAMD, our data set contained multiple missing data points. To account for this, we utilized the missMDA R package by Josse and Husson (2016) that analyzes incomplete data sets for underlying data structures. We also performed an imputation of the missing data values and reanalyzed the data set using missMDA to confirm the data structure. Figures 2A,B were generated using Sigma Plot ver. 11.0 (Systat Software, Inc.) and R.

## Results and Discussion

### Study Location and Number

Leaf removal (early and late) has been studied as an approach for mitigating major wine grape production issues for ~2 decades (Figure 2). The first studies on this topic were conducted in the late 1980s and early 1990s and focused on application at the fruit-set stage (E-L 27) (Coombe, 1995) when fruit are ~4–6 mm in diameter. In 1988, Bledsoe et al. were the first to show that leaf removal at fruit set could increase sugar concentrations (total soluble solids, TSS) in fruit while decreasing acidity in California's dry climate (Bledsoe et al., 1988). Soon after, additional publications reported that this practice performed at the same timing greatly decreased the incidence of *Botrytis cinerea* (English et al., 1989; Gubler et al., 1991). Given that disease pressure is higher in more humid climates, researchers in these regions sought to understand whether performing this practice earlier (pre-bloom) to alter cluster architecture could further reduce bunch rot disease. This is reflected by the number of studies focusing on the pre-bloom timing occurring more recently in the last 10–15 years (Figure 2). With our data curation steps considered, Poni et al. were the first to characterize the response of pre-bloom leaf removal using the “Trebbiano” cultivar in a peer-reviewed journal (Poni et al., 2006). They revealed that this practice significantly reduced bunch rot incidence and increased total soluble solids (TSS) concentrations in the fruit at harvest.

**Figure 3:**
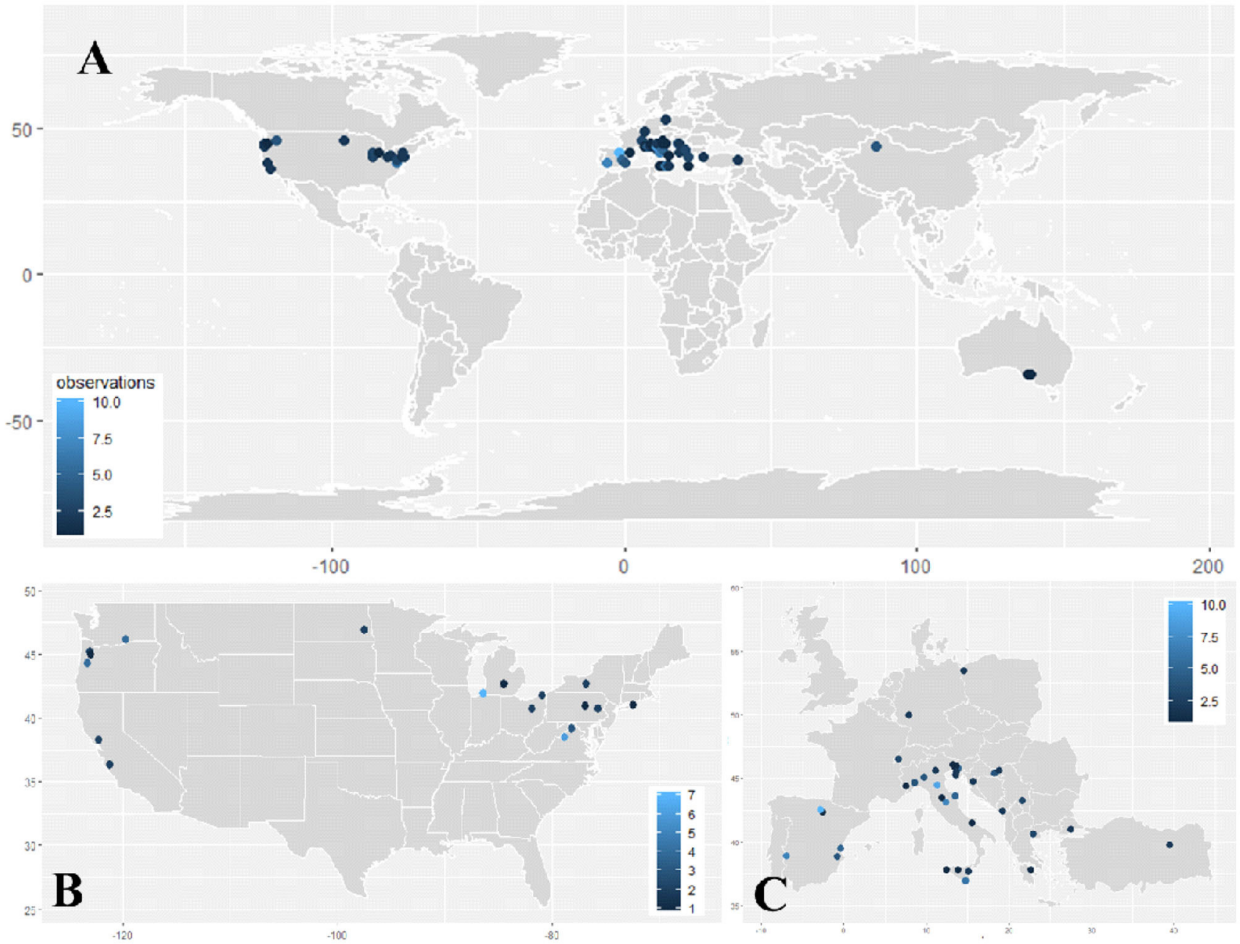
Dot plot heatmap depicting the location of studies meeting meta-analysis criteria (Table 1) in the **(A)** world, **(B)** United States of America, and **(C)** Europe. Heatmaps represent the number of experimental observations included from each location.

Leaf removal implemented prior to (or during) bloom has now been tested in many growing regions throughout the world (Figure 3A), with the majority of studies being conducted in the United States, Italy, and Spain (Figures 3B,C). Since the mid-2000s, multiple researchers have thoroughly tested this approach in growing regions, which are represented in Figure 3. These include the following: Ollauri (La Rioja) and Badajoz (Extremadura), Spain; Bologna, Perugia and Ragusa, Italy; Benton Harbor, Michigan; Northwest Oregon (Willamette Valley); and Shenandoah Valley, Virginia. With the exception of Badajoz (Climate 1) and Perugia (Climate 2), these growing regions share a similarity of producing wine grapes in an environment receiving low accumulation of heat units (GDD) and/or environments receiving high volumes of precipitation (Climates 1 and 3) (Supplementary Table 1, Supplementary Figure 1). This is reflective of two major objectives for performing pre-bloom leaf removal: reducing bunch rot disease and enhancing fruit ripening.

### Effect of PB Leaf Removal on Production Parameters

**Figure 4:**
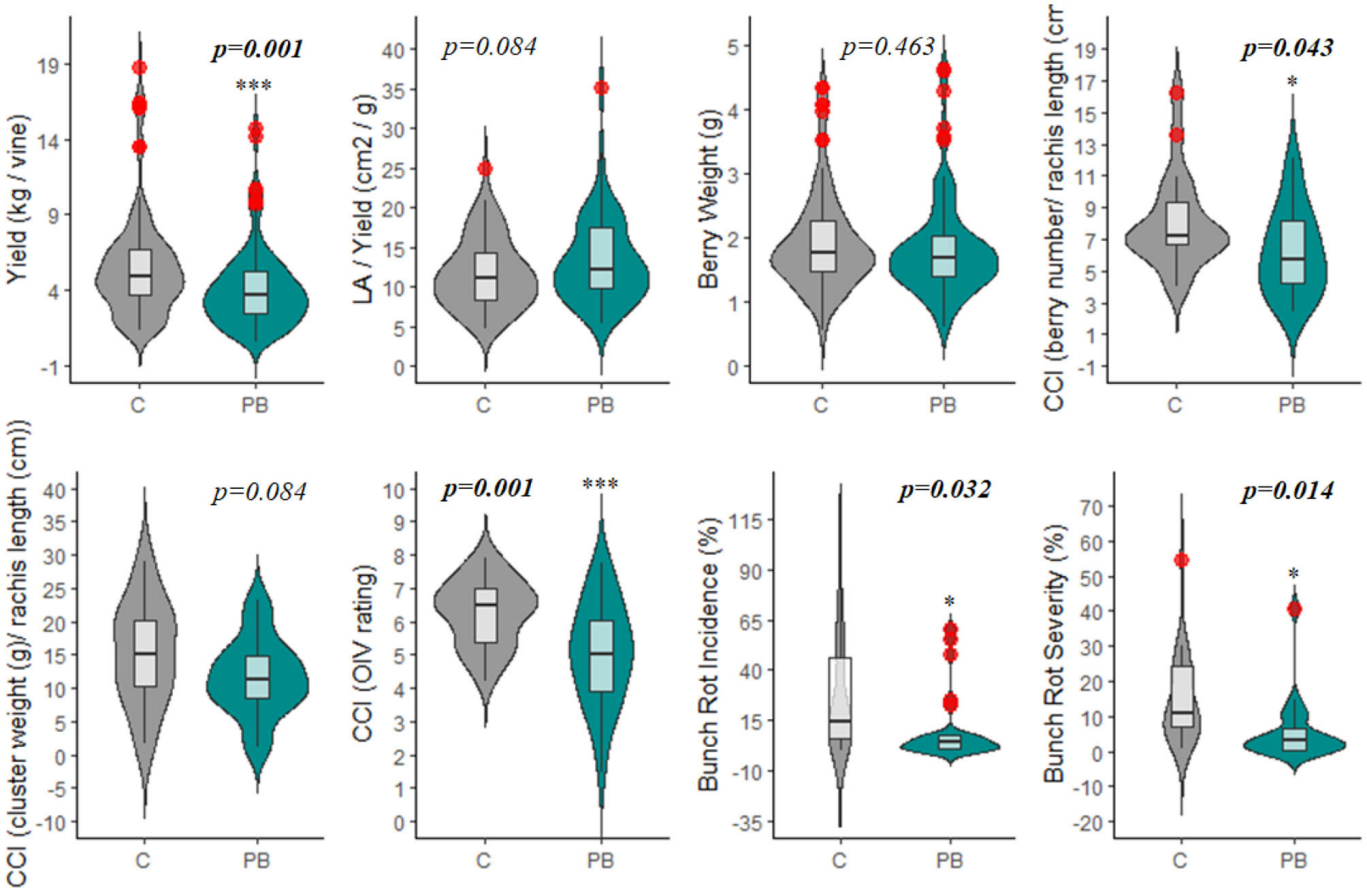
Violin plot displaying the impacts of pre-bloom leaf removal on yield, vine balance (LAY), cluster compactness, and bunch rot parameters. Red circles represent significant outliers in data sets. C, non-defoliated control; PB, pre-bloom leaf removal treatment; CCI, cluster compactness index; LA, leaf area. *p < 0.05; ***p < 0.001.

The leaf area removed from plants with the PB treatment ranged from 30.7 to 96.0%, with an average of 61.6% (data not reported). Although there is a large variation in floret sensitivity to abscission among grape cultivars (Lebon et al., 2004), PB led to a significant reduction in yield per vine (Figures 4, **6**, Supplementary Table 2). This is due to the decrease in fruit set that occurs when a large percentage of the carbohydrate source (leaves) is removed from the plant during the period of strong vegetative growth, drastically reducing the carbon portioning to the reproductive organs (Frioni et al., 2018). The decrease in fruit set corresponded to a significant reduction in yield (26%) in response to the PB treatment (Figure 4). In Table 1, yield is highlighted as a production parameter having one of the most consistent alterations by PB, at 80%. The similarity in yield reduction from a wide range of percentage of leaf area removed is due to translocation of carbohydrates from shoots having a surplus of carbohydrates to those with a deficit to support fruit set (Frioni et al., 2019). This suggests that the leaf area of the whole vine is important for dictating fruit set and yield reduction and not just the leaf area of individual shoots.

**Table 1:** Listing of parameters, variable type, and number of observations (comparing C and PB).

**Acronym**	**Parameter**	**Variable**	**Total observations^**a**^**	**Significant observations^**b**^**
**DEPENDENT VARIABLES**				
Yield	Yield (kg/vine)	Production	103	82 (80%)
LAY	Leaf area/Yield (cm^2^/g)	Production	62	19 (31%)
BW	Berry weight (g)	Production	97	39 (40%)
CCI1	Cluster Compactness Index (berry number/cm^2^)	Production	20	10 (50%)
CCI2	Cluster Compactness Index [berry weight (g/cm^2^)]	Production	19	13 (68%)
CCI3	Cluster Compactness Index (OIV visual rating)	Production	33	27 (82%)
BRI	Bunch rot incidence (%)	Production	26	16 (62%)
BRS	Bunch rot severity (%)	Production	20	12 (60%)
TSS	Total soluble solids (°Brix)	Fruit quality	108	56 (52%)
pH	pH	Fruit quality	102	25 (25%)
TA	Titratable acidity (g/L)	Fruit quality	105	34 (32%)
ANT1	Total anthocyanins (mg/100 g) FW skins	Fruit quality	14	7 (50%)
ANT2	Total anthocyanins (mg/100 g) FW berry	Fruit quality	73	44 (60%)
PHE1	Total phenolics (mg/100 g) FW skins	Fruit quality	15	8 (53%)
PHE2	Total phenolics (mg/100 g) FW berry	Fruit quality	53	34 (64%)
PHE3	Total phenolics (Absorbance Units)	Fruit quality	12	4 (33%)
**CATEGORICAL VARIABLES**				
BC	Berry color	–	136	–
CL	Climate	–	136	–
CUL	Cultivar	–	136	–
RS	Rootstock	–	123	–
VA	Vine age (years)	–	121	–

a Number of observations comparing between C and PB.

b *Number of observations where PB was significantly larger or smaller (p < 0.05) than C*.

Also relating to the reduction in fruit set, Cluster Compactness indices (CCI2, CCI3) were significantly decreased at a high rate of 68 and 82%, respectively (Table 1). Meanwhile, CCI1 reported only 50% of observations as significantly altered (Table 1). The differences observed in CCI parameters suggest varying sensitivities of the indices for detecting fruit-set alteration and, consequently, modifications of morphological characteristics of the clusters. Although CCI3 was the most sensitive among the indices at detecting modifications to cluster morphology, this method is highly subjective; fruit compactness is visually matched to a 6-point scale. Therefore, we suggest that CCI2 should be utilized in future studies that measure this parameter, as it is both a more sensitive metric than CCI1 and a more rapid approach. CCI describes the “openness” of the cluster, which is greatly enhanced as a result of floret abscission (Tello and Ibáñez, 2018). This decrease in compactness positively impacts the quality of fruit, as an “open” cluster is more resistant to bunch rot disease (Table 1 and Figure 4) (Hed et al., 2009). Wind speed through the fruit zone is increased by three to four times after PB leaf removal (English et al., 1989). As a result, the evaporative potential of water from the fruit surface is higher, preventing water from collecting on the fruit surface (Acimovic et al., 2016). This is the reason for the consistent reduction (62 and 60%) in bunch rot incidence (BRI) and bunch rot severity (BRS), respectively. The identical rate of significant observations for both BRI and BRS highlight the viability of either parameter as a suitable index for estimating changes in bunch rot disease infection (Table 1 and Figures 4, **6**). In addition to significantly decreasing BRI and BRS, PB leaf removal greatly narrowed the distribution of the data when compared to the non-defoliated control (C), the undefoliated treatment (Figure 4). This suggests that a threshold exists whereby continuing to decrease fruit set has no additional impact on lowering disease pressure.

### Effect of PB Leaf Removal on Fruit Quality Parameters

**Figure 5:**
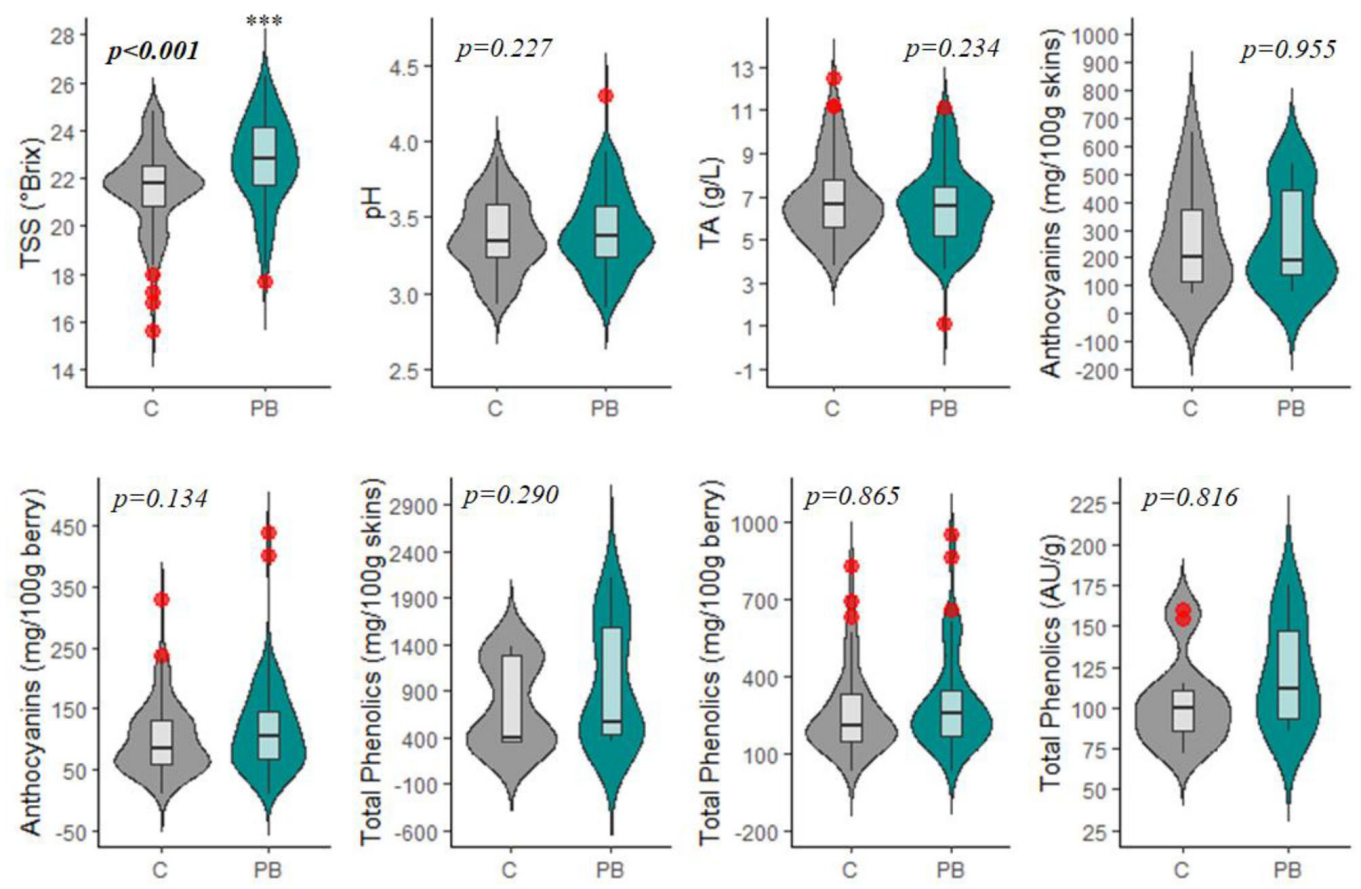
Violin plot displaying the impacts of pre-bloom leaf removal on basic fruit quality parameters, total anthocyanins, and total phenolics. Red circles represent significant outliers in data sets. C, non-defoliated control; PB, pre-bloom leaf removal treatment; TSS, total soluble solids; TA, titratable acidity. ***p < 0.001.

**Figure 6:**
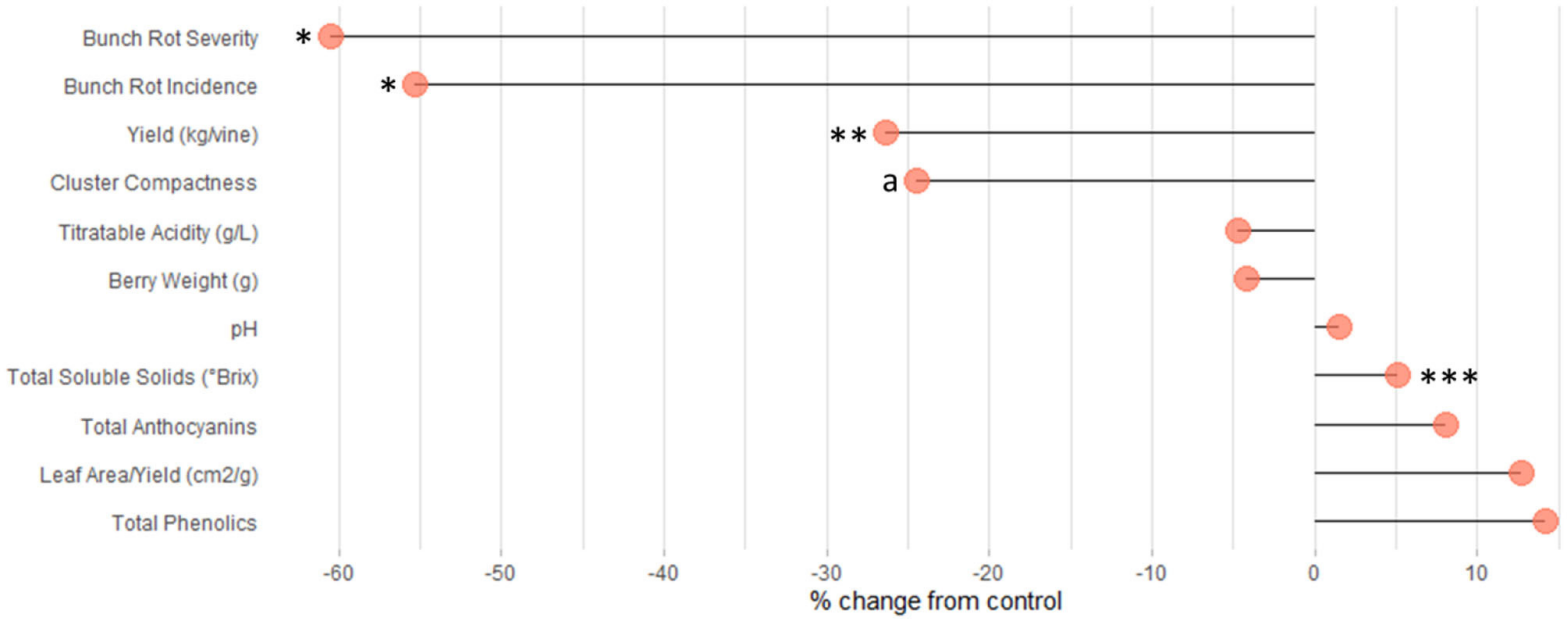
Lollipop plot visualizing the percent change in dependent variables in PB compared to C. a, CCI, two of three parameters representing this value are significant. *p < 0.05; **p < 0.01; ***p < 0.001.

**Figure 7:**
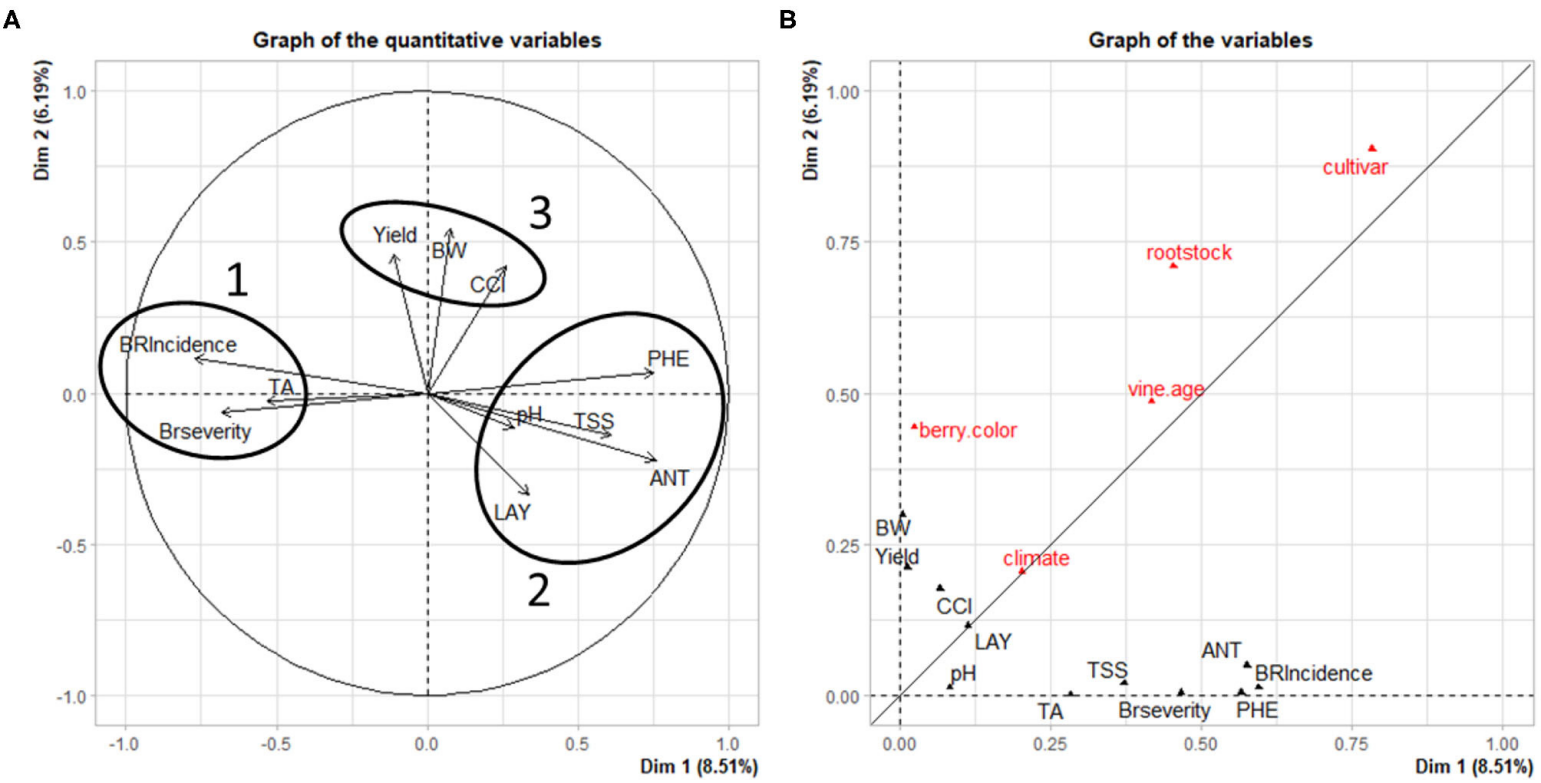
**(A)** Principal component analysis (PCA) displaying the relationship between the percent change of the dependent variables in response to PB and **(B)** PCA variables visualizing the relationships between categorical and the % change of the dependent variables in response to PB. Full names for parameter acronyms are available in Supplementary Table 2.

Most studies focusing on pre-bloom leaf removal (PB) prioritize basic fruit quality components (TSS, pH, TA) over that of secondary metabolite parameters (ANT, PHE) (Table 1). TSS was the only quality parameter that reported a significant change in response to PB treatments (Figure 5, Supplementary Table 2). This could be attributed to the significant decrease in yield or bunch rot disease (Figures 4, 6, Supplementary Table 2). However, the combination of multiple factors is likely to drive the increase in fruit sugar concentration at harvest reported by the studies. In this meta-analysis, TSS increase was not shown to be explicitly related to the yield reduction (Figure 7A), as has been suggested in some studies (Xi et al., 2018). Instead, decreased yield promotes a greater ratio between leaf area and yield (LAY), which has been used an index of vine balance, shown to be more related to fruit quality parameters than vine crop level in several previous studies (Kliewer and Dokoozlian, 2005; Pastore et al., 2011; Sivilotti et al., 2020). This is also the case here in our elaboration of data from the available literature (Figure 7A). It is worth noting that the increase in LAY is not solely due to the decrease in yield. Numerous studies show that removing leaves prior to bloom in the fruit zone leads to a stimulation of lateral leaf growth, leading the significantly larger leaf area in PB vines at harvest (Poni et al., 2006, 2009). Although LAY was increased by 12.7%, it was not significantly altered from C (Figures 4, 7A, Supplementary Table 2). This is likely related to the variability and inconsistency among the methods used to calculate the leaf area partitioning of this metric. Additionally, the contribution of decreased BRI and BRS to increasing TSS is realized in this study (Figure 7A). However, it is challenging to explicitly link these parameters, as one form (sour rot) decreases sugar concentrations, while the other (gray mold) increases it (VanderWeide et al., 2020), and it was not possible to distinguish between both forms of bunch rot in this analysis.

Interestingly, PB leaf removal altered secondary metabolites, namely, anthocyanins (ANT) and phenolics (PHE), to a greater percent than TSS (Figure 6) but were not significantly modulated from the C (Figures 5, 6, Supplementary Table 2). This is likely due to the large variability that exists in total anthocyanin and phenolic concentrations between cultivars (Mattivi et al., 2006), as well as the many different extraction protocols and chromatography/spectroscopy methods used for the quantification of the metabolites (De Beer et al., 2004). Specifically, ANT1, ANT2, PHE1, PHE2, and PHE3 had 9-, 41-, 4-, 72-, and 10-fold differences in concentrations between the smallest and largest data points, respectively.

In many studies, ANT and PHE concentrations were expressed in both mg/tissue and mg/berry. In the case of ANT and PHE, calculation on a mg/berry basis (ANT2, PHE2) resulted in a more consistent alteration following the PB treatment than measurement on a per-tissue basis (ANT1, PHE1) (Table 1). Grape phenolics are thought to be impacted by berry size; however, this has not been firmly established (Walker et al., 2005; Ariani et al., 2016). Most phenolic compounds are located in the skin or seed tissues, and smaller berries have a greater ratio of skin and seeds to pulp and therefore will contribute more anthocyanins and phenolics per volume of fruit (Roby et al., 2004). However, berry weight (BW) was not decreased significantly in this experiment, suggesting that this slight increase in ANT and PHE in response to PB is due to an increased biosynthesis (Pastore et al., 2013) or, in the case of anthocyanins, increased skin thickness (Poni et al., 2009; Verdenal et al., 2019).

## Relationship Between Categorical and Dependent Parameters

In Figure 7A, the principal component analysis (PCA) displays relationships among dependent variables analyzed in this work. Three distinct groups of variables are visible; two exhibit an inverse relationship to one another on dimension 1, while the third is along the positive axis of dimension 2. In group 1, bunch rot parameters (BRI, BRS) are closely aligned with TA. This could be due to cluster sour rot infection increasing acetic acid concentrations (Zoecklein et al., 1995), which would influence TA by increasing it. However, multiple studies included in this analysis did not distinguish between either form of bunch rot disease (sour rot, gray mold), making this difficult to confirm. An additional explanation is that a higher TA, indicative of under-ripe fruit, is an artifact of fruit being harvested early due to high presence of either sour rot of gray mold in fruit. This is backed up by the near-opposite relationship between groups 1 and 2.

Group 2 includes TSS and pH, which increase in ripening grapes, opposite to TA, which decreases. In addition to TSS and pH, group 2 also includes the other quality parameters: ANT and PHE. During the ripening process, sugars are understood to be a physiological “trigger” for the accumulation of ANT (Larronde et al., 1998; Lecourieux et al., 2014), which likely explains the grouping of these two parameters. This is not the case for most phenolics (PHE); however, anthocyanins comprise the majority of this group post-veraison, suggesting that PHE is reflective of ANT. Group 2 also indicates a relationship between fruit quality parameters and LAY. It is well-known that this ratio, often referred in viticulture as “vine balance” index, rather than the simple reduction of yield, influences fruit quality parameters (Kliewer and Dokoozlian, 2005; Parker et al., 2015). This is supported by yield in group 3, which, along with BW and CCI, were not advertently related with parameters from either group 1 or 2.

Regarding group 3, the positive relationship between BW (berry weight) and CCI (number of berries per cluster) on yield is unsurprising, as the number and size of individual berries directly influence yield. However, the lack of a strong relationship between CCI and BW with the other groups in Figure 7A is worth noting. Our previous research identified a significant negative relationship between cluster compactness and ANT concentration in “Merlot” berries (VanderWeide et al., 2018), while others have confirmed this with additional quality metabolites (Ziegler et al., 2020). Likewise, cluster compactness has been shown to correlate negatively with bunch rot parameters (Marois et al., 1986; Hed et al., 2009). This lack of a relationship between CCI and either bunch rot or fruit quality parameters may be due to two reasons. First, and only regarding CCI and bunch rot disease, the presence of many observations deriving from warm and hot regions that display low bunch rot disease pressure may be skewing the data sets for BRI and BRS. Second, for both relationships, it may be that other factors have a greater influence on these parameters, such as the aforementioned one between LAY, TSS, and ANT, or an open cluster zone for bunch rot parameters, as is mentioned in the literature (English et al., 1989; VanderWeide et al., 2020). The underlying genetic and physiological mechanisms governing BW are complex (Dai et al., 2011), and PB did not cause a consistent modulation to them, different from other grapevine cultural practices (Gambetta et al., 2020). A reduction in BW by PB was reported only following the removal of 10 leaves from vines (Acimovic et al., 2016). At this threshold, the limitation of source availability was likely extended through the more active phase of cell division. Our analysis restricts studies to those that removed five to eight leaves. Additionally, BW was significantly increased and decreased from the control in different observations within this analysis, which likely explains why BW was not correlated to either bunch rot disease or fruit quality parameters.

The second component of Figure 7 reveals the relationships among the categorical and dependent variables from each study. All categorical variables were similarly affected by both dimensions with the exceptions of berry color and rootstock, which were more closely aligned on dimension 2. Yield and BW were also oriented along dimension 2. With regards to yield and berry color, this is likely due to the different cropping (yield adjustment) standards for white vs. red grapes, as red cultivars require greater GDD to reach harvest maturity and therefore require a more aggressive reduction in yield when compared to white cultivars, especially in cooler climates. Berry weight is also related to berry color, as red cultivars tend to have smaller berries than white cultivars. The primary roles of rootstock selection are to control water uptake and vine growth (Poni et al., 2017). The relationship between rootstock and these production parameters is intriguing, as there is no subsequent impact on quality parameters.

Surprisingly, Climate had the smallest effect among categorical variables on dependent variables. Meanwhile, Cultivar and Rootstock had the greatest influences on these variables. This is, in part, due to the fact that most cultivars and rootstocks are selected on a climate-specific basis (Keller, 2015), therefore mitigating differences in climate among growing regions. Additionally, red cultivars are known to possesses higher concentrations of total phenolics than white cultivars, and white cultivars almost exclusively lack anthocyanin production (Mattivi et al., 2006). The lesser influence from climate may also come from scales of data between the climatological data and the leaf removal experiments. The climatology data were taken as 30-year climate norms for each site, while the studies were taken from certain years' worth of data. Higher resolution climate data—weather data taken for each year of study for all 59 studies—would likely increase the connection with climate. However, because of a lack of quality weather data in certain study areas, this was not possible. Future work with higher resolution data may yet reveal a stronger connection. This suggests the need for further investigation into our data set to more explicitly uncover the influence of climate and other categorical variables on the “success” of PB.

## Conclusion

This meta-analysis was conducted using 59 publications that describe the response of grapevines to pre-bloom leaf removal: an important grapevine canopy management technique. The results of this work provide a clear physiological picture into the response of PB on both production and fruit quality parameters. Pre-bloom leaf removal applied early in the vine growth and developmental stages restricts carbohydrate availability to inflorescence, which accelerates inflorescence abscission and causes a reduction in fruit set. This significantly decreases yield by 26%. Additionally, lowered fruit set significantly reduced CCI, which, in turn, led to a reduction in bunch rot incidence (BRI) and severity (BRS) by ~55–60%. Among fruit quality parameters, only °Brix was significantly increased by PB, likely influenced by both the decrease in yield and bunch rot disease. PCA indicated a strong relationship between the percent increase in vine balance (leaf-to-fruit ratio) and TSS in response to PB. This analysis also revealed a strong correlation between the percent increase in multiple fruit quality parameters, including TSS, pH, anthocyanins, and phenolics; the latter two are likely influenced by the higher TSS. Together, this study provides grape producers with a clear outline of the benefits of performing pre-bloom leaf removal to achieve high fruit quality in challenging growing climates.

## Data Availability Statement

The data in this article is available on request to the corresponding author.

## Author Contributions

JV and PS planned and organized the study. JV, PS, and EN curated the literature data. JV and CG elaborated the data, ran the statistical analysis, and arranged the tables and figures. JV, PS, and CG wrote the first draft of the manuscript. SP critically revised the manuscript. All authors contributed to the article and approved the submitted version.

## Conflict of Interest

The authors declare that the research was conducted in the absence of any commercial or financial relationships that could be construed as a potential conflict of interest.
