# Supplementary material for: Impacts of Pre-bloom Leaf Removal on Wine Grape Production and Quality Parameters: A Systematic Review and Meta-Analysis
Source: Front Plant Sci. 2021 Feb 4;11:621585. doi: 10.3389/fpls.2020.621585 (PMC7889588; doi:10.3389/fpls.2020.621585)
Supplement: Supplementary file 1 [file Table_1.docx]

**Supplementary Table 1.** Climatic classification of observations used in the analysis.

| **Class #** | **Classification** | **Region/City** | **Country** | **Climate Data Source** |
| --- | --- | --- | --- | --- |
| 1 | Hot | Badajoz | Spain | NCDC |
| 1 | Hot | Podgorica | Montenegro | Hydrological and Meteorological Service of Montenegro |
| 1 | Hot | Guadajira | Spain | NCDC |
| 1 | Hot | Podunavlje | Croatia | Datameteo |
| 1 | Hot | Foggia | Italy | Datameteo |
| 1 | Hot | Moixent | Spain | Agencia Estatal de Meteologia |
| 1 | Hot | Udine | Italy | NCDC |
| 1 | Hot | Resuttano | Italy | NCDC |
| 1 | Hot | University of Adelaide | Australia | NCDC |
| 2 | Warm / Dry | Tebano | Italy | Datameteo |
| 2 | Warm / Dry | Prosser | USA | NCDC |
| 2 | Warm / Dry | Erzincan | Turkey | NCDC |
| 2 | Warm / Dry | Tekirdağ | Turkey | NCDC |
| 2 | Warm / Dry | Zafferana Etnea | Italy | Datameteo |
| 2 | Warm / Dry | Valencia | Spain | Agencia Estatal de Meteologia |
| 2 | Warm / Dry | Requena | Spain | Agencia Estatal de Meteologia |
| 2 | Warm / Dry | Barossa Valley | Australia | NCDC |
| 2 | Warm / Dry | Prokuplje | Serbia | NCDC |
| 2 | Warm / Dry | Central Po Valley | Italy | Datameteo |
| 2 | Warm / Dry | Ancona | Italy | Datameteo |
| 2 | Warm / Dry | Mandi´cevac | Croatia | Datameteo |
| 2 | Warm / Dry | West Po Valley | Italy | Datameteo |
| 2 | Warm / Dry | Arezzo | Italy | Datameteo |
| 2 | Warm / Dry | Perugia | Italy | Datameteo |
| 2 | Warm / Dry | Hormilla | Spain | NCDC |
| 2 | Warm / Dry | Torre del Colle | Italy | Datameteo |
| 3 | Warm / Wet | Colli Piacentini | Italy | Datameteo |
| 3 | Warm / Wet | Benton Harbor | USA | NCDC |
| 3 | Warm / Wet | Bologna | Italy | NCDC |
| 3 | Warm / Wet | Val d’Illasi | Italy | Datameteo |
| 3 | Warm / Wet | Shenandoah Valley | USA | NCDC |
| 3 | Warm / Wet | Winchester | USA | NCDC |
| 3 | Warm / Wet | Andreas | USA | NCDC |
| 3 | Warm / Wet | Pully | Switzerland | MeteoSwiss |
| 3 | Warm / Wet | Lewisburg | USA | NCDC |
| 3 | Warm / Wet | Oslavio | Italy | Datameteo |
| 3 | Warm / Wet | Castelvecchio | Italy | Datameteo |
| 3 | Warm / Wet | Slap | Slovenia | NCDC |
| 4 | Cool | Salem | Oregon | NCDC |
| 4 | Cool | Dayton | Oregon | NCDC |
| 4 | Cool | Ollauri | Spain | NCDC |
| 4 | Cool | Koreniki | Croatia | NCDC |
| 4 | Cool | Potoce | Slovenia | NCDC |
| 4 | Cool | Szczecin | Poland | NCDC |
