# Supplementary material for: Impacts of Pre-bloom Leaf Removal on Wine Grape Production and Quality Parameters: A Systematic Review and Meta-Analysis
Source: Front Plant Sci. 2021 Feb 4;11:621585. doi: 10.3389/fpls.2020.621585 (PMC7889588; doi:10.3389/fpls.2020.621585)
Supplement: Supplementary file 2 [file Table_2.docx]

**Supplementary Table 2.** Impact of pre-bloom leaf removal on dependent variables.

| **Parameter** | **C^b^** | **PB** | ***p*-value** |
| --- | --- | --- | --- |
| ***Production Parameters*** |  |  |  |
| Yield (kg/vine) | 5.71 ± 3.70^a^ | 4.18 ± 2.70 | ***0.001*** |
| Leaf Area/Yield (cm2/g) | 11.6 ± 4.48 | 13.3 ± 5.41 | *0.070* |
| Berry Weight (g) | 1.93 ± 0.766 | 1.84 ± 0.773 | *0.389* |
| Cluster Compactness Index (berry number/length (cm^2^)) | 8.10 ± 2.96 | 6.21 ± 2.73 | ***0.043*** |
| Cluster Compactness Index (cluster weight (g)/length (cm^2^)) | 15.5 ± 7.54 | 11.7 ± 5.78 | *0.085* |
| Cluster Compactness Index (OIV visual rating) | 6.28 ± 0.976 | 5.02 ± 1.53 | ***<0.001*** |
| Bunch Rot Incidence (%) | 24.6 ± 26.0 | 10.9 ± 17.3 | ***0.031*** |
| Bunch Rot Severity (%) | 15.0 ± 12.8 | 5.92 ± 9.26 | ***0.014*** |
| ***Fruit Quality Parameters*** |  |  |  |
| Total Soluble Solids (°Brix) | 21.4 ± 2.21 | 22.4 ± 2.07 | ***<0.001*** |
| pH | 3.39 ± 0.251 | 3.44 ± 0.285 | *0.226* |
| Titratable Acidity (g/L) | 6.79 ± 1.89 | 6.50 ± 1.93 | *0.280* |
| Total Anthocyanins (mg/100g) FW skins | 268 ± 183 | 272 ± 172 | *0.955* |
| Total Anthocyanins (mg/100g) FW berry | 100 ± 59.7 | 117 ± 76.2 | *0.134* |
| Total Phenolics (mg/100g) FW skins | 755 ± 457 | 973 ± 937 | *0.290* |
| Total Phenolics (mg/100g) FW berry | 383 ± 938 | 413 ± 865 | *0.865* |
| Total Phenolics (Absorbance Units/g) | 151 ± 187 | 170 ± 215 | *0.816* |

^a^Data are expressed as means ± standard deviation.

^b^C, control; PB, pre-bloom leaf removal.
