# Supplementary material for: Impacts of Pre-bloom Leaf Removal on Wine Grape Production and Quality Parameters: A Systematic Review and Meta-Analysis
Source: Front Plant Sci. 2021 Feb 4;11:621585. doi: 10.3389/fpls.2020.621585 (PMC7889588; doi:10.3389/fpls.2020.621585)
Supplement: Supplementary file 3 [file Data_Sheet_1.docx]

**Supplementary Figure 1.** Categorization of observations based on climatic conditions (daily temperature, precipitation).
